# Rare retinal complications of bone marrow transplantation (BMT): a case report

**DOI:** 10.1186/s12886-018-0855-6

**Published:** 2018-09-14

**Authors:** Farhat Butt, Martin McKibbin

**Affiliations:** Leeds Teaching Hospital, Beckett St, Leeds, LS9 7TF England

**Keywords:** Bone marrow transplant, Retinopathy, Ciclosporin, Retinal toxicity

## Abstract

**Background:**

Bone marrow transplantation retinopathy is a rare condition affecting the posterior pole. The purpose of this case report is to highlight the possible risk factors and clinical features.

**Case presentation:**

A 19y old male with relapsed and refractory acute lymphoblastic leukaemia was admitted under haematology with pyrexia of unknown origin. At the time of his admission, he reported bilateral and sequential visual impairment for 2 days. On examination, there was bilateral profound retinopathy across the posterior poles. This was symmetrical and with associated macular oedema. Infective aetiology was excluded and cyclosporine was stopped. Although no definitive treatment was initiated the visual acuity improved whilst macular oedema fluctuated.

**Conclusion:**

BMT and chemotherapy can cause ocular complications but these are usually confined to the anterior segment. Posterior segment complications in the form of retinopathy is very rare. We report this case to highlight some of the clinical features and course of disease.

## Background

Acute lymphoblastic leukaemia (ALL) is a malignancy of the lymphoid progenitor cells in the bone marrow, blood and extra medullary sites. It affects both children and adults with a peak prevalence between the ages of 2 and 5 years [1, 2]. It is the most common childhood malignancy [3]. With medical advances the survival rates have improved to around 90% [2]. Approximately 60% of children are in complete remission at 5 years from initial diagnosis.^3^ In adults the prognosis is far less favourable [4, 5].

In the past few decades the treatment protocols have been extensively revised and the main treatment is chemotherapy [4, 5]. BMT is an established treatment for high-risk patients and patients with relapsed or refractory disease [4]. Before infusing the bone marrow patients receive high-dose chemotherapy, this may be an combined with total body irradiation [6]. The aim of this is to destroy host malignant tissue and minimise rejection [6].

Treatment can lead to ocular complications such as keratoconjunctivitis sicca, cataracts and conjunctival graft-v-host disease (GVHD) [6, 7]. Keratoconjunctivitis sicca may lead to corneal problems such as microbial keratitis and therefore requires prompt diagnosis and treatment. GVHD can result in chronic disease which can be challenging to manage.

Rarely BMT results in posterior segment problems and often these maybe of infective origin or due to cytotoxic medication such as cyclosporin [7, 8]. BMT can also lead to non- progressive retinopathy with macular oedema however this is usually a diagnosis of exclusion. The aetiology of BMT retinopathy is thought to be multifactorial [9] and patients tend to have favorable visual outcomes with no sequelae. There is no established treatment for this condition and the retinopathy can resolve spontaneously [9]. Here we report a case of BMT associated retinopathy in a young man.

## Case presentation

A 19y old male with relapsed and refractory ALL was admitted under haematology with pyrexia of unknown origin. At the time of his admission, he reported sequential, bilateral visual impairment that had started 2 days previously. There was no accompanying redness, pain, photophobia, photopsia or floaters. His past medical history involved total body irradiation, chemotherapy and, 4 months previously BMT to treat the ALL. There was no previous ocular or family history of note. His medication included cyclosporine to prevent BMT rejection, and prophylactic posaconazole and acyclovir. At the time of eye clinic review his bloods were Hb- 8.2 g/dL, plt-18 × 10 ^9^ /L and WCC- 0.1 × 10^9^/L. PCR from peripheral blood for viral DNA tested negative for EBV, CMV and ADV.

**Fig. 1:**
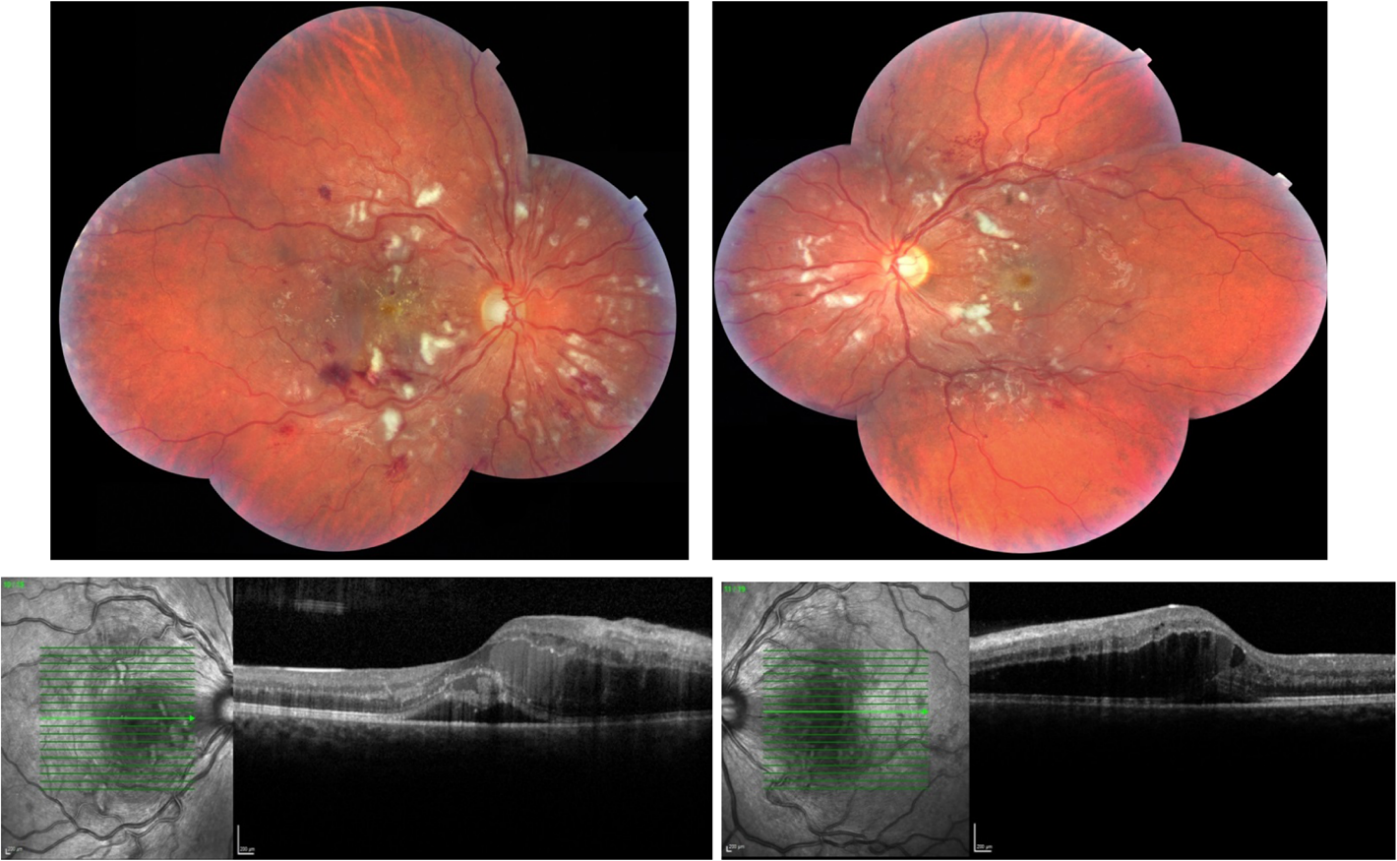
Fundus photographs and macular OCT images showing the extent of retinopathy

On examination Snellen acuity was reduced to 6/36 on the right and 6/24 on the left. The anterior segments were normal. There was no anterior chamber (AC) activity or vitritis and the intraocular pressures were normal. Dilated fundus examination showed bilateral and symmetrical retinopathy with cotton wool spots and retinal haemorrhages across the posterior pole with relative sparing of the peripheral retina. Cystoid macular oedema in both eyes, with sub-retinal fluid in the right eye, was noted on optical coherence tomography (OCT) imaging. The central sub-field thickness (CST) was 557 μm and 603 μm in the right and left eyes. Imaging findings are shown below in the colour fundus pictures and OCT images (Fig. 1).

### Management

Given the pyrexia of unknown origin and the above fundus findings an urgent AC tap was arranged to exclude viral retinitis. This was negative for CMV, HSV, VZV and EBV DNA. Given the persistent visual impairment but without an obvious infective aetiology, cyclosporine was stopped under haematology guidance, as this can be retinotoxic, and 40 mg of oral steroids commenced.

Vision subjectively started to improve over the subsequent 3–4 days despite no resolution of cystoid macular oedema. Two weeks after presentation, the CST was 815 μm in the right eye and 1016 μm in the left eye despite an improvement in VA. Oral steroids were slowly tapered down and stopped over a period of 4 weeks. The cyclosporine was not restarted. The vision continued to improve to 6/9 in the right and 6/6 in the left accompanied by the resolution of macular oedema over several months. This improvement did not coincide with haematological normalisation.

Sadly over the course of the subsequent few months, the patient’s systemic health deteriorated and he died as a result of pulmonary complications.

## Discussion

Organ transplant is more likely to result in ocular complications involving the anterior segment such as keratoconjunctivitis sicca, conjunctival graft-versus-host disease and superficial punctate erosions [6, 7, 10]. Keratoconjunctivitis sicca is a potential complication in around 50% of patients typically presenting 6 months after transplantation [10].

Posterior segment complications are fortunately rare but visually significant. A study based in a tertiary referral unit reported posterior segment complications to be as high as 12.8% [8] The exact aetiology of BMT retinopathy has not been defined but can be divided broadly into three categories; infection, microvasculopathy and haematological abnormalities [7]. Histopathalogical studies confirm an ischaemic process with capillary endothelial loss but no obvious sequel [9]. The visual prognosis is generally good with resolution of retinopathy and restoration of vision in few months as highlighted in this case [7, 8].

BMT retinopathy is a sight threatening bilateral and symmetrical disease of a multifactorial origin. It tends to occur within the first 6 months of transplantation. Contributing factors include toxicity e.g. from cyclosporine, or high dose chemotherapy, severe haematological abnormality, total body irradiation and infection e.g. toxoplasmic retinochoroiditis although this is reported to be very rare [7, 8]. Literature suggests complete resolution within a few months without any neovascularisation but little is known about treatment [8, 9].

## Conclusion

To summarise, we report a very rare case of BMT retinopathy in a patient with refractory ALL. Our patient presented with poor central vision and was found to have retinopathy with associated macular oedema. During the follow up period the vision started to improve despite fluctuating macular oedema and without any definitive treatment. There is limited literature on the subject and thus our understanding of the disease remains incomplete. We hope that by reporting this case we can highlight the typical features associated with BMT retinopathy.

## Abbreviations

AC: Anterior chamber
ADV: Adenoviral
ALL: Acute lymphoblastic leukaemia
BMT: Bone marrow transplantation
CMV: Cytomegalovirus
CST: Central sub-field thickness
EBV: Epstein-Barr virus
GVHD: Graft versus host disease
Hb: Haemoglobin
HSV: Herpes simplex virus
OCT: Optical coherence tomography
Plt: Platelets
VA: Visual acuity
VZV: Varicella zoster virus
WCC: White cell count
